# Flexibility in Spanish Elite Inline Hockey Players: Profile, Sex, Tightness and Asymmetry

**DOI:** 10.3390/ijerph17093295

**Published:** 2020-05-09

**Authors:** Antonio Cejudo, Víctor Jesús Moreno-Alcaraz, Riccardo Izzo, Francisco Javier Robles-Palazón, Pilar Sainz de Baranda, Fernando Santonja-Medina

**Affiliations:** 1 Department of Physical Activity and Sport, Faculty of Sport Sciences, Regional Campus of International Excellence “Campus Mare Nostrum”, University of Murcia, 30100 Murcia, Spain; antonio.cejudo@um.es (A.C.); victorjm@um.es (V.J.M.-A.); franciscojavier.robles1@gmail.com (F.J.R.-P.); 2 Dipartimento di Scienze Biomolecolari, Scuola di Scienze Motorie, Università degli Studi, 61029 Urbino, Italy; riccardo.izzo@uniurb.it; 3 Sports and Musculoskeletal System Research Group (RAQUIS), University of Murcia, 30100 Murcia, Spain; fernando@santonjatrauma.es; 4 Department of Surgery, Pediatrics, Obstetrics and Gynecology, Faculty of Medicine, Regional Campus of International Excellence “Campus Mare Nostrum”, University of Murcia, 30100 Murcia, Spain

**Keywords:** range of motion, lower limb, stretching, roller hockey

## Abstract

Limited ranges of motion (ROM) have been considered as a relevant risk factor for team sports injuries. The main purposes of the current study were to describe the lower extremity ROM profile, muscular tightness and asymmetries in elite inline hockey players and to examine sex-related differences. Twenty professional inline hockey players from 2 Spanish National Teams (male and female) were measured of passive hip extension [HE], hip adduction with hip flexed 90° [HAD-HF], hip flexion with the knee flexed [HF-KF] and extended [HF-KE], hip abduction with the hip neutral [HAB] and hip flexed 90° [HAB-HF], hip external [HER] and internal [HIR] rotation, knee flexion [KF], ankle dorsiflexion with the knee flexed [ADF-KF] and extended [ADF-KE] ROMs of the dominant and non-dominant leg ROMs were taken. A paired *t*-test was carried out to assess asymmetries. ROM values were classified as “normal versus tightness”, and “normal versus asymmetry” according to the proposed reference values. The effect size for each variable was analyzed. Male team exhibited asymmetry in HF-KF (133.2° dominant vs. 129.8° non-dominant; *p* = 0.042; d = 0.7243 [moderate effect sizes]) and female team in ADF-KF (38.8° dominant vs. 41.0 non-dominant; *p* = 0.001; d = 0.6 [moderate effect sizes]) and HAB ROM (41.2° dominant vs. 38.8 non-dominant; *p* = 0.005; d = 1.1767 [moderate effect sizes]). Male players reported asymmetry in HAD-HF (n = 5), HER (n = 4) and HE (n = 3), whereas female players presented asymmetries in HER (n = 4), HE (n = 3) and KF (n = 2). Overall, 20–100% of all participants showed limited KF, HF_KE, HIR, HE, ADF_KF, HAD-HF, HF-KF, ADF_KE, HTR and HER ROM. The results of this study reinforce the requirement of prescribing exercises aimed at improving hip, knee and ankle ROM within everyday inline hockey practices. In addition, as some asymmetries were found, unilateral flexibility training should be considered where appropriate.

## 1. Introduction

Measuring a range of motion (ROM) is a clinical procedure to evaluate a mechanical joint problem caused by disorders of the locomotor system. The purpose of ROM measurement is to observe the extent of inhibition, but also to identify the factors that restrict joint movement and to evaluate the effectiveness of treatment and training [1]. The measurement method of ROM used internationally was established by the American Academy of Orthopedic Surgeons [2] and American Medical Association [3], and since 1963, both institutions have provided the reference average values for the normal joint. These reference values have been the most commonly used resource by health and sports professionals to mark quantifiable goals in the training of flexibility in healthy individuals and athletes with or without pathology [3,4,5,6,7,8]. Nevertheless, the measurements have not been consistently stratified by sport, age and sex. 

When ROM values have been evaluated in sport, it has been observed a great variation between different sports [9] and even within the same sport, depending on joint and movement [7], sex [9,10], age [11], competitive level [12,13], lateral dominance (dominant and non-dominant) [14], and/or tactical position [15,16]. Likewise, different studies have shown that the restriction in these ROM values could negatively affect sports performance [17,18,19] and increase the injury risk [20]. The limited hip extension ROM (<13°) have been related to ankle sprains and thigh muscle strains in senior soccer players [21], as well as to rectus abdominal muscle strains [22] in professionals tennis players; limited ankle dorsiflexion ROM may also predispose to patellar tendinopathy (<45°) in volleyball players [23] and basketball players (<36.5°) [24]. In ice-hockey players, limited hip abduction ROM (<67.5°) have been related to groin injuries [25].

Therefore, it remains essential to establish an individual standard flexibility profile where the optimum ROM value in each joint for a specific sport, promoting the higher physical-technical sports performance of the player with a lower predisposition to sports injury [26]. This flexibility profile might help coaches with setting specific and quantified objectives in flexibility training during their daily basis routines.

Inline hockey is an intermittent high-intensity team sport characterized by continuous skating actions produced at different velocities, directions and durations [27,28]. In Spain, the number of players involved in this sport has increased significantly; over the last 10 seasons, an increase of almost 60% in the number of inline hockey licenses has been observed [29]. In order to successfully compete at the international level, athletes must be able to meet the high demands hockey places on both the oxidative and glycolytic energy systems. Athletes should also have the strength, power and flexibility to meet the agility and technical skill required to skate, shoot and pass the puck [30]. 

In this sense, the increase of ROM values, especially in the iliopsoas, adductors, hamstring, quadriceps and surae triceps, might improve skating efficiency and speed, and other puck handle skills [31,32,33], favoring the players’ performance and injury risk reduction [20,30]. Although some studies have analyzed the range of motion (ROM) in several team sports, most of them have been focused on football [11,34], futsal [10,16,26] or handball [35] players. The scientific evidence according to hockey players’ ROM values is scarce, and strictly limited to the ice hockey modality [25,36,37]. However, it should be noticed the evident differences in skating motions between ice and inline hockey modalities [38], which supports the necessity of establishing a specific ROM profile for each one. We hypothesize that the players analyze in this study, which corresponds to the international elite of hockey, should show higher ROM scores than general population reference values [2,3,4,5,6]. We also expect to find differences in ROM values related to the sex of players as have been previously described for other sports [9,10]. 

Therefore, the main purposes of the current study were, firstly, to describe the lower limb ROM profile in inline hockey players and to determine sex-related differences, and, secondly, to identify individual players with limited ROM values and bilateral asymmetries.

## 2. Materials and Methods 

### 2.1. Participants

The participants were recruited from the last pre-competition meeting of the Spanish National Team before the Roller Games World Championship in Nanjing (2017).

Before data collection, participants completed a questionnaire containing questions about their sport-related background (tactical position, current competitive level, dominant lower extremity, sport experience), anthropometric characteristics [age, body mass, stature and body mass index], and training regimen (weekly practice frequency, hours of inline hockey practice per week and day, resting periods, types of fitness and training load). 

Data from questionnaires reported that the sample was homogeneous in potential confounding variables, except in height and training months/year (Table 1). None of the participants was involved in systematic and specific stretching regimes in the last 6 months. In addition, the participants do not usually perform stretching exercises daily during their pre-exercise warm-up and post-exercise cool down phases.

The exclusion criterion was a history of orthopedic problems to the knee, thigh, hip, or lower back in the last 3 months due to the residual symptoms could have an impact on the habitual players’ movement competency and/or lower extremity ROM profile. The study was conducted at the end of the competition phase of the year 2017. 

Before any participation, experimental procedures and potential risks were fully explained to the participants in verbal and written form, and written informed consent was obtained. The experimental procedures used in this study were following the Declaration of Helsinki and were approved by the Ethics and Scientific Committee of the University of Murcia (Spain) [ID: 1702/2017].

### 2.2. Testing Procedure

**Figure A1:**
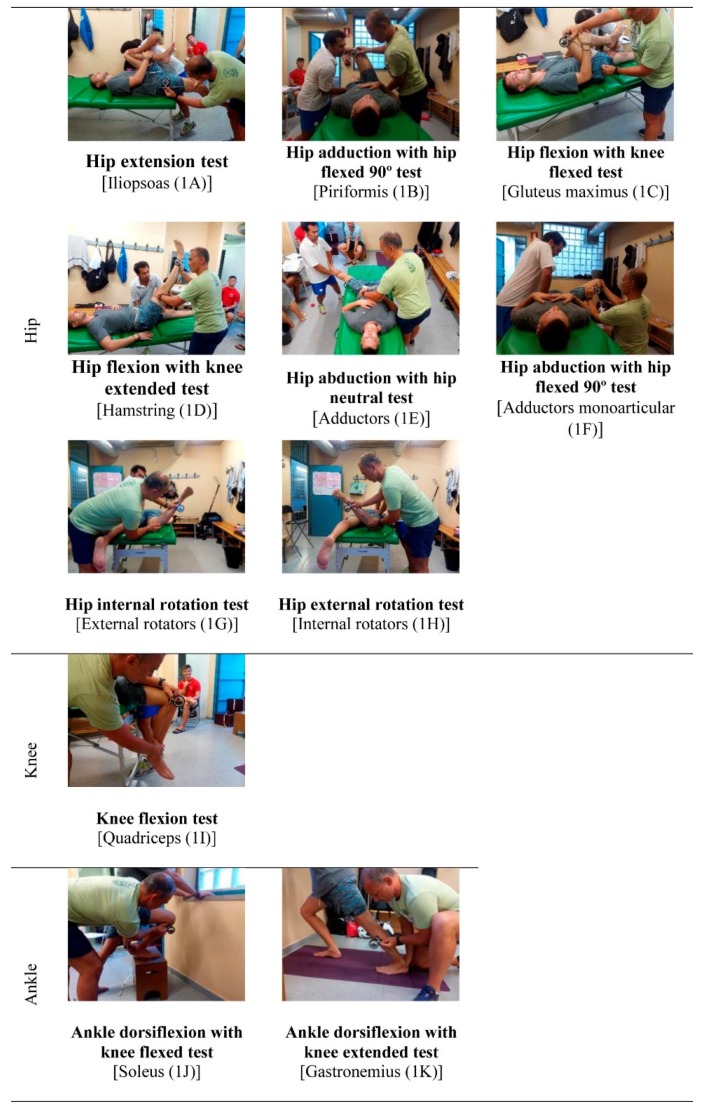
Lower extremity ranges of motion of the “ROM-SPORT” protocol. Hip extension test [HE] (**1A**); hip adduction with hip flexed 90° test [HAD-HF] (**1B**); hip flexion with knee flexed test [HF-KF] (**1C**); hip flexion with knee extended test [HF-KE] (**1D**); hip abduction with hip neutral test [HAB] (**1E**); hip abduction with hip flexed 90° test [HAB-HF] (**1F**); hip internal rotation test [HIR] (**1G**); hip external rotation test [HER] (**1H**); knee flexion test [KF] (**1I**); ankle dorsiflexion with knee flexed test [ADF-KF] (**1J**); ankle dorsiflexion with knee extended test [ADF-KE] (**1K**).

The passive hip extension [HE], hip adduction with hip flexed 90° [HAD-HF], hip flexion with knee flexed [HF-KF] and extended [HF-KE], hip abduction with hip neutral [HAB] and hip flexed 90° [HAB-HF], hip external [HER] and internal [HIR] rotation, knee flexion [KF], ankle dorsiflexion with knee flexed [ADF-KF] and extended [ADF-KE] ROMs of the dominant and non-dominant leg were assessed following the methodology previously described [10] (Appendix A, Figure A1).

These tests were selected because they have been considered appropriate by American Medical Organizations, AAOS [2] and AMA [3] and included in manuals of Sports Medicine and Science [4,5,6,29,30]; The content validity is determined by judging if an instrument or procedure accurately measures and represents the variable of interest [39]. In this sense, all the selected assessment tests for the ROM-SPORT have been considered appropriate by the American Medical Organizations [2,3] and included in the accredited manuals of Sports Medicine and Science [4,5,6,7,8], based on anatomical knowledge and extensive clinical experience. In addition, studies from our laboratory have reported moderate to high intra-examiner reliability for the procedures employed (variability ranging from 4° to 7°) [40,41]. 

One week before the start of the study, all the inline hockey players completed a familiarization session with the purpose of knowing the correct technical execution of the exploratory tests by means of the practical realization of each one of them. The dominant leg was determined objectively by checking the participant´s preferred kicking limb. All tests were carried out by the same two sport scientist experts under stable environmental conditions.

Prior to the testing session, all participants performed the dynamic warm-up designed by Taylor, Sheppard, Lee & Plummer [42]. The overall duration of the entire warm-up was approximately 20 min. After the warm-up, inline hockey players were instructed to perform, in a randomized order, two maximal trials of each ROM test for each leg, and the mean score for each test was used in the analyses. Inline hockey players were examined wearing sports clothes and without shoes. A 30 s rest was given between trials, legs, and tests. 

For the measurement, an ISOMED Unilevel inclinometer (Portland, Oregon) was used with an extendable telescopic rod [2], a metal goniometer with long arm (Baseline^®^ Stainless) and “lumbosant” -lumbar support- to standardize the lumbar curvature [43,44]. Before each assessment session, the inclinometer was calibrated to 0° with either the vertical or horizontal. The angle between the longitudinal axis of the mobilized segment was recorded (following its bisector) with the vertical or the horizontal [3,10]. Regarding the assessment of hip abduction movement, a metal goniometer of the long arm (Baseline^®^ Stainless) was used.

One or both of the following criteria determined the endpoint for each test: (a) an examiner palpable or appreciated some compensation movement that increased the ROM onset of pelvic rotation, and/or (b) the inline hockey player feeling a strong but tolerable stretch, slightly before the occurrence of pain [10].

### 2.3. Statistical Analysis

Prior to the statistical analysis, the distribution of raw data sets was checked using the Shapiro-Wilk tests to determine normal distribution. The results demonstrated that all data had a normal distribution. Descriptive statistics including means and standard deviations were calculated for hip, knee and ankle ROM measures separately by leg (dominant and non-dominant). 

Data were analyzed using independent sample Student’s t-test to examine possible differences in demographic variables and hip, knee and ankle ROMs between the males and females group. In addition, paired t-test was carried out to assess differences between the values of the dominant and non-dominant sides. Additionally, Cohen’s effect size was calculated for all results, and the magnitudes of the effect were interpreted according to the criteria of Hopkins, Marshall, Batterham & Hanin [45] in which the effect sizes less than 0.2, from 0.2 to 0.59, from 0.6 to 1.19, from 1.20 to 2.00, from 2.00 to 3.99 and greater than 4.00 were regarded as trivial, small, moderate, large, very large and extremely large, respectively. The authors arbitrarily chose moderate as the minimal relevant effect level with practical application in the results. 

To help the practical interpretation of the results obtained, the ROM values presented in the current study were classified as “normal versus tightness”, and “normal versus asymmetry” according to the proposed reference values to consider that an athlete is more prone to suffer an injury. In these movements where the sport-specific cut-off score was not found (HAD-HF, ADF_KE, HER, HIR, HAB-HF, HF), the reference value for the general population was used to categorize players with limited ROM. Otherwise, when several cut-off scores were found for the same ROM, the most conservative criteria were selected. All the cut-off scores used in this study are presented in Table 2. Likewise, the cut-off scores to identify asymmetries in ROM used were 6 and 10 degrees, depending on the kind of ROM value (6° for lowest ROM scores –HE, HAB-HF, ADF-KE, ADF-KF and HAB– and 10° for highest ROM scores –HER, HIR, HAB-HF, HF-KE, KF and HF-KF) [14,15,16,17,18,19,20,21,22,23,24,25,26,27,28,29,30,31,32,33,34,35,36,37,38,39,40,41,42,43,44,45,46].

Finally, an independent sample T-test was used to determine differences between mean values in both normal and tightness groups. Effect size for each variable was analyzed throughout Pearson (r) statistic (0.0–0.39 = low, 0.4–0.69 medium, and 0.7–1 = high effect [51]). All the statistical analysis were conducted by the SPSS software (Statistical Package for Social Sciences, v. 24.0, for Windows; SPSS Inc., Chicago, IL, USA), establishing the minimum significance level of 5%.

## 3. Results

To address the specificity of flexibility in sport, four goalkeepers (2 males and 2 females) and 2 male junior inline-hockey players were excluded. Finally, twenty players met the inclusion and exclusion criteria (Table 1). Among the variables that were assessed before the beginning of the study, the only significant difference detected between the groups (male vs. female) was in height (male group 1.73 m vs. female group 1.66 m; *p* = 0.001) and training months/year (man group 11.2 months vs. female group 10 months; *p* = 0.003).

Regarding bilateral ROM asymmetries, significant differences were found by gender. A high number of asymmetries in both males and females were found in HER (n = 4) with higher non-dominant values than the dominant limb and HE (n = 3) with higher dominant limb values than non-dominant. However, male inline hockey players showed the highest number of asymmetries in HAD-HF (n = 5) and only one player with HAD-HF asymmetry was found in females. Individual analysis by gender reported significant differences in HF-KF ROM asymmetries for males (d = 0.7243 [moderate]) and HAB ROM asymmetries (d = 1.1767 [moderate]) for female inline hockey players (Table 3 and Table 4).

Independent sample t-test showed significant differences (*p* < 0.05) by sex in the HAD-HF (dominant limb), ADF-KE (dominant limb), HIR (non-dominant limb), HER (dominant limb), HAB-HF (both limb), KF (dominant limb) and HF-KF (both limb) ROM. Female inline hockey players exhibited higher ROM in HAD-HF (31.2° vs. 25°; *p* = 0.007; d = −1.35 [large]), HIR (39.2° vs. 37.2°; *p* = 0.030; d = −1.05 [moderate]), HER (64.8° vs. 57.6°; *p* = 0.016; d = −1.19 [moderate]), HAB-HF (73° vs. 64,8°; *p* = 0.001; d = 1.35 [large]), KF (115.4° vs. 102°; *p* = 0.007; d = −1.41 [large]) and HF-KF (141° vs. 131.5°; *p* = 0.005; d = 1.68 [large]) than male inline hockey players. Male hockey players exhibited greater ADF-KE (36.2° vs. 31°; *p* = 0.045; d = 1.00 [Moderate]) ROM than females.

Limited range of motion in both dominant and non-dominant extremities was found for 20 players in KF and HF_KE (100%), 19 players in HIR and HAB_HF (95%), 18 players in HE (90%), 16 players in ADF_KF (80%), 11 players in HAD-HF (55%), 7 players in HF-KF (35%), 5 players in ADF_KE (25%), and 2 players in HER (10%) ROMs (Table 5 and Table 6). 

## 4. Discussion

**Figure 1:**
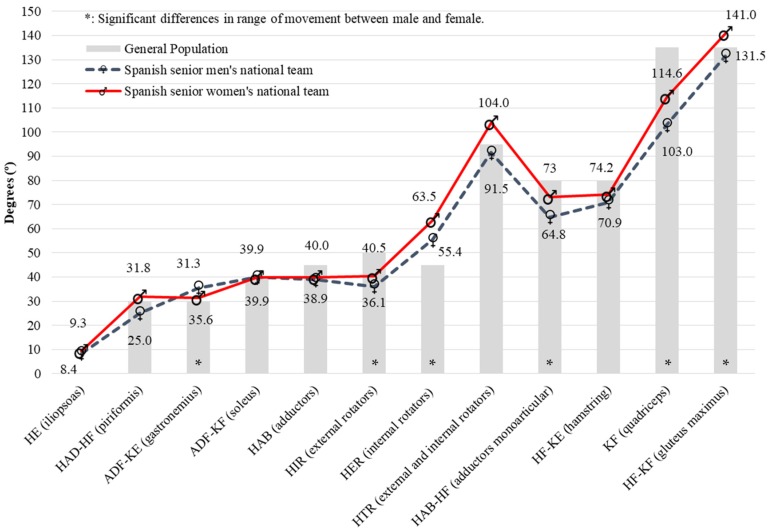
Flexibility profile differences between male and female elite inline hockey players, and general population. Average ROM values of both dominant and non-dominant extremities are presented.

The main purpose of this study was to describe the lower limb ROM profile in inline hockey players and to examine sex-related differences. To accomplish this goal, we collected bilateral passive ROM measurements of the hip, knee and ankle joints from a sample of individuals without known medical or physical conditions affecting the joint mobility. A dynamic stretch warm-up was applied before the ROM assessment because: (1) all the tests required a large muscle tension stimulus; (2) to lessen the effects of muscle lengthening from repeated trials during data collection; and (3) to reduce the variability and standard error of measurements by minimizing the effect of different muscle temperature on muscle flexibility [52,53]. The flexibility profile of the 20 inline-hockey players of each Spanish National Team, which has been determined with the results of the 11 ROM-SPORT protocol measures, is shown in the Figure 1. When these results are analyzed, our primary findings showed certain specific differences in the flexibility profiles of elite inline hockey players according to general population, sex, tightness and bilateral asymmetries (side-to-side).

On the one hand, inline-hockey players (mean values of the male and female samples) exhibited greater HIR (14°), HE (9°) and ADF-KE (3°) ROM than general population [2,6,47]. Likewise, higher values to the general population have been observed in HAD-HF (6.8°) and HF-KF (6°) ROM in female inline hockey players (Figure 1). It seems that the physical and technical demands in this sport (for example, HIR in phase of stride swing recovery and stride crossover side, HE in phase of stride push-off and ADF-KE in phase of stride push-off) involve dynamic movements with greater ROM than walking or running tasks, favoring an increase of the muscular extensibility and ROM [32]. If a player is unable to fully extend their rear leg during skating, it will decrease skating speed, leading to poor skating mechanics and resulting in a subsequent further loss of flexibility [30]. Thus, the increment of flexibility, especially in the iliopsoas, piriformis and gastrocnemius might improve skating speed, power and efficiency, as well as the different technical skills of inline hockey [20,31,33]. On the other hand, the Spanish inline-hockey players showed lower ROM values than general population in KF (26°), HER (12°), HAB-HF (11°), HF-KE (7°) and HAB (6°) ROMs. Forward propulsion is resulted from summating extension, abduction and rotation of the hip, extension of the knee, and plantar flexion of the ankle [32]. However, due to the design of the hockey skate, the joint ankle has a limited ROM, indicating that propulsion is resulted primarily from knee extension and hip abduction and extension [20,32]. Therefore, the primary leg muscles involved in propulsion are quadriceps, abductors and gluteus, with the gluteus maximus being primarily responsible for the majority of the power production during push-off [20,32]. The quadriceps muscles are most active when extending the knee during the propulsion phase and the hamstring muscles are most involved during the gliding phase [20,32]. The strong concentric and eccentric loads on these muscles have the potential to generate muscle damage that, without adequate measures and recovery time, could induce alterations in the mechanical and neuronal properties of the muscle-tendon units, including a reduction of normal ROM [54]. Similar results are observed in ADF-KF (0°) and ADF-KE (3°) due to the restriction of ankle range of motion by the high cut boot and rigid sole [32]. 

Taking into account the specificity of the flexibility, it would be necessary to compare the results of the present study with data of the same sport, sex and competitive level. However, no previous studies have been found that address the assessment of angular flexibility in inline-hockey players. Our results can only be discussed with two scientific papers, which assessed the ROM in several movements in ice hockey modality [36,37]. In the 16 college ice hockey players assessed in the study published by Wilcox et al. [37] higher values were observed in comparison to the results presented by males in this study in HE (24.3° vs. 8.4°), HAD-HF (27.1° vs. 25°), HAB (44.5° vs. 38.9°) and HF-KE (99.9° vs. 70.9°) ROMs, respectively, while much lower values were observed in the HIR (28.1° vs. 36.1°) and HER (28.9° vs. 55.4°) ROMs. The research of Tyler, et al. [36] also showed higher values, and similar to the scores reported by Wilcox et al. [25], in the HAB ROM (45.8° vs. 38.9°) in comparison to this study for 47 professional ice hockey players. Perhaps, the greater professionalization of ice hockey might explain these results. It is plausible that the higher competitive level of ice hockey players may entail an increase in the number of stretching exercises and myofascial release techniques included in their daily training routines, favoring a better performance in ROM measurements. The inclusion of both interventions as part of sports training has previously demonstrated the increase in post-intervention muscle extensibility [55,56]. Contrarily, the low ranges observed in the rotation of the hip in the ice hockey players could be a consequence of the more intense overloads of the hip rotator muscles that stabilize the hip, since the ice modality might require greater demands of strength, speed and aggressiveness [57].

As expected, sex affects the results of flexibility. Women hockey players displayed greater HAD-HF, HIR, HER, HAB-HF, KF and HF-KF ROMs and smaller ADF-KE ROM than men players; in the rest of the movements, similar values have been found (Figure 1). These findings cannot be compared with the previous reports about the effects of the hockey modality on flexibility [25,36,37,58], because in these previous studies the differences according to the participants’ sex were not evaluated. However, there are kinematic studies [54,57,59,60] on skating start propulsion and transition from start to maximum speed, which showed sex-specific differences in terms of velocity progression, as well as hip and knee joint angles. In particular, males demonstrated greater net positive acceleration during the initial accelerative steps, and greater hip abduction and knee flexion (~10°) from ice contact to push off [59]. The results of the present study disagree with this justification. In these two movements, we found that female players have highest values (> 8°) than male players (ROM HAB-HF [73° vs. 64.8°] and KF [114.6° vs. 103°]). This fact can be explained by a greater muscular volume of the gluteus maximus and quadriceps in men, which limits both movements during the impulse phase. The results of this study could have implications in the design of flexibility training according to gender differences to improve physical and technical sports performance.

A second aim was to identify individual players with limited ROM values and bilateral asymmetries. When a more in-depth and individual analysis was carried out (classification “normal vs. tightness” based on the cut-off values provided by previous investigations), the results of the present study showed a very high number of inline hockey players (between 20–100% of the total players assessed) with muscle tightness in all movements, with the exception of the ABD ROM. The accumulation of sports training and match loads (13.5 years of experience, 10.6 training months/year and 5.2 training hours per week) of the participants in this study could be behind the predominance of muscle tightness displayed by the inline hockey players. In this sense, the repetitive actions that require short stride lengths and high stride frequencies to increase the speed can derive in muscle ROM reductions [20,32], which can also be favored by the absence of stretching routines at the beginning and at the end of the training and competitive sessions (confirmed by a self-reported questionnaire completed by the players involved in this study). Several authors have shown the importance of optimal flexibility in the hip, knee and ankle that allows greater knee flexion, hip flexion and ankle plantar flexion prior to push-off and keeping a greater forward lean of the trunk to increase the speed of skating [20,31]. However, this flexibility may not only be necessary to inline hockey performance; several injuries have been associated with reductions in ROMs, such as knee sprains, groin strains and low back pain [61,62]. Indeed, previous studies have shown that these injuries might represent the most frequent types of injury in inline-hockey players [38,61]. Therefore, the great percentage of players with ROM limitations found in the current research may also be related to the high incidence of injury reported by inline hockey players compared to other athletes [62].

Another risk factor that predisposes to sports injury is the bilateral asymmetry of flexibility [14,63]. The statistical analysis only showed asymmetry of flexibility with a moderate effect size (*p* ≤ 0.005; d ≥ 0.6) in the HF-KF ROM in the male national team and in ADF-KF and HAB ROMs in the female team. However, the individual analysis identified a greater number of players with asymmetry in males (n = 19) than females (n = 15) in all movement assessed. The movements with the highest number of players with asymmetry were HIR (n = 8), HE (n = 6), HAD-HF (n = 6), KF (n = 4), ADF-KE (n = 2), HAB (n = 2), HER (n = 2), HAB-HF (n = 2) and HF-KF (n = 2) ROMs. Although the technical execution of this sport is symmetrical [59,60,64], unilateral muscle adaptations were found. Ice hockey skills include the general movement patterns of skating, stick handling, and checking [59,60,64]. It seems that the players have a greater ability with the dominant or non-dominant limb in each of the phases of the stride (glide, push off and recovery). Therefore, it seems important to analyze the possible specific adaptations of inline hockey in all ROMs of the lower extremities, in both grassroots and elite groups. The flexibility profile determined in this study can be used by sports and health professionals as a reference of the best ROM values in this sport, which could help to establish objectives in order to improve the physical-technical sport performance of young inline hockey players. Whereas, the values of tightness can be used to identify the players that present a greater risk of injury and, later, plan effectively and establish successful training programs. 

According to the results of present study, after the evaluation of the ROM with the ROM-SPORT protocol, the players classified with muscle tightness (iliopsoas, piriformis, triceps surae, muscles external and internal rotators, monoarticular adductors, hamstring, quadriceps and gluteus maximus) should participate in a systematic stretching program. In order to design an efficient and safe stretching program, the following indications should be taken into account: a) meet the minimum stretch exercise recommendations proposed by The American College of Sports Medicine (weekly stretching frequency, volume and intensity), (b) using the appropriate stretching technique for each part of the training (warm up, main activity and cold down), (c) feeling of muscle stretching during exercise, and (d) avoid compensatory movements during the technical execution of exercises.

Future prospective studies should be designed to determine the cut-off values that discriminate the predisposition of injury in this sport, and thus better understand the weight of muscle tightness on the risk of sports injury.

## 5. Conclusions

The flexibility profile of the 10 male inline hockey players assessed in the current study indicate 8.4° for the HE, 25° for the, 35.6° for the ADF-KE, 39.9° for the ADF-KF, 38.9° for the HAB, 36.1° for the HIR, 55.4° for the HER, 64.8° for the HAB-HF, 70.9° for the HF-KE, 103° for the KF and 131.5° (133.2° dominant limb, 129.8° non-dominant limb) for the HF ROMs.

The flexibility profile of the 10 female inline hockey players evaluated indicate 9.3° for the HE, 31.8° for the HAD-HF, 31.3° for the ADF-KE, 39.9° (38.8° dominant limb, 41° non-dominant limb) for the ADF-KF, 40.5° (41.2° dominant limb, 38.8° non-dominant limb) for the HAB, 40.5° for the HIR, 63.5° for the HER, 73° for the HAB-HF, 74.2° for the HF-KE, 114.6° for the KF and 141° for the HF ROMs.

Significant differences (*p* < 0.05) according to sex were observed in the HAD-HF, ADF-KE, HIR, HER, HAB-HF, KF and HF-KF ROM. 

The results of this work could have implications in the design of flexibility training with respect to gender differences to improve physical-technical sports performance. Additionally, it is recommended the inclusion of stretching exercises or the increase of the training load in these muscle groups, in order to achieve or maintain normal values and symmetry of flexibility, and consequently, decrease the risk of sports injury in the assessed muscles. 

## Figures and Tables

**Table 1:** Demographic and sport data of the Spanish National Selection inline-hockey players ^a^.

Variable	Male (n = 10)	Female (n = 10)	Total (n = 20)
Age (years)	22.30 ± 2.54	22.70 ± 3.33	22.50 ± 2.89
Body mass (kg)	71.87 ± 9.64	66.83 ± 9.20	69.35 ± 9.53
Height (m) *	1.73 ± 0.05	1.66 ± 0.05	1.69 ± 0.06
BMI (kg/m2)	23.96 ± 2.77	24.32 ± 2.88	24.14 ± 2.76
Years of experience	14.20 ± 2.97	12.90 ± 3.98	13.55 ± 3.49
Training months/year *	11.20 ± 0.92	10.00 ± 0.00	10.60 ± 0.88
Training days/week	2.80 ± 0.42	2.80 ± 1.03	2.80 ± 0.77
Training hours per week	5.40 ± 2.27	5.00 ± 1.83	5.20 ± 2.02

^a^ Values are expressed as mean ± standard deviation; BMI: body mass index; * significant differences between men and female were observed in height and training months/year (p ≤ 0.003).

**Table 2:** Published cutoff reference values used to identify muscle tightness.

**ROM**	**HE [21]**	**HAD-HF [3,8]**	**ADF-KE [6,47]**	**ADF-KF [23]**	**HAB [2]**	**HIR [6,48]**
**Value**	13°	30°	30°	45°	28°	45°
**ROM**	**HER [3]**	**HAB-HF [2]**	**HF-KE [49]**	**KF [50]**	**HF-KF [3]**	
**Value**	50°	80°	88°	132°	135°	

ROM: range of movement; Hip extension test [HE]; hip adduction with hip flexed 90° test [HAD-HF]; ankle dorsiflexion with knee flexed test [ADF-KF]; ankle dorsiflexion with knee extended test [ADF-KE]; hip abduction with hip neutral test [HAB]; hip internal rotation test [HIR]; hip external rotation test [HER]; hip abduction with hip flexed 90° test [HAB-HF]; hip flexion with knee extended test [HF-KE]; knee flexion test [KF]; hip flexion with knee flexed test [HF-KF].

**Table 3:** Passive maximum range of motion values of 10 inline hockey players of the Spanish elite male team.

ROM	Dominant	Non-Dominant	Players with Asymmetries	*p*-Value	Cohen’s d
HE (iliopsoas)	7.0 ± 5.8°	9.80 ± 7.8°	3	0.132	−0.3288 Small
HAD-HF (piriformis)	25.0 ± 3.3°	27.60 ± 5.8°	5	0.128	−0.4851 Small
ADF-KE (gastronemius)	36.2 ± 5.5°	35.0 ± 6.4°	1	0.279	0.1811 No effect
ADF-KF (soleus)	39.6 ± 4.9°	40.2 ± 5.7°	0	0.591	−0.2209 Small
HAB (adductors)	39.2 ± 3.2°	38.60 ± 3.5°	1	0.560	0.3333 Small
HIR (external rotators)	35.0 ± 6.6°	37.2 ± 4.6°	1	0.146	−0.3922 Small
HER (internal rotators)	57.6 ± 10.9°	53.2 ± 8.1°	4	0.068	0.4417 Small
HAB-HF (monoarticular adductors)	64.0 ± 7.2°	65.6 ± 6.1°	1	0.387	−0.1534 Small
HF-KE (hamstrings)	71.4 ± 4.2°	70.4 ± 4.1°	0	0.322	0.25 Small
KF (quadriceps)	102.0 ± 9.6°	104.0 ± 14.4°	2	0.430	−0.1699 Small
HF-KF (gluteus maximus)	133.2 ± 6.3°	129.8 ± 5.5°	1	0.042	0.7243 Moderate

Hip extension test [HE]; hip adduction with hip flexed 90° test [HAD-HF]; ankle dorsiflexion with knee flexed test [ADF-KF]; ankle dorsiflexion with knee extended test [ADF-KE]; hip abduction with hip neutral test [HAB]; hip internal rotation test [HIR]; hip external rotation test [HER]; hip abduction with hip flexed 90° test [HAB-HF]; hip flexion with knee extended test [HF-KE]; knee flexion test [KF]; hip flexion with knee flexed test [HF-KF]; The magnitude of the effect size of the pooled Standardised mean differences (SMD) was interpreted as trivial or no effect if SMD <0.2; small if SMD 0.2 to 0.59; moderate if SMD 0.6 to 1.19; large if SMD 1.20 to 2.00; very large if SMD 2.00 to 3.99 and extremely large if SMD greater than 4.00.

**Table 4:** Passive maximum range of motion values of 10 hockey players of the Spanish elite female national team.

ROM	Dominant	Non-Dominant	Players with Asymmetries	*p*-Value	Cohen’s d
HE (iliopsoas)	8.2 ± 5.9°	10.4 ± 4.4°	3	0.178	−0.4417 Small
HAD-HF (piriformis)	31.2 ± 5.6°	32.4 ± 4.7°	1	0.279	−0.2209 Small
ADF-KE (gastronemius)	31.0 ± 5.4°	31.6 ± 6.8°	1	0.685	0 No effect
ADF-KF (soleus)	38.8 ± 5.6°	41.0 ± 5.3°	0	0.083	−0.56 Small
HAB (adductors)	41.2 ± 2.9°	38.8 ± 3.2°	1	0.005	1.1767 Moderate
HIR (external rotators)	41.8 ± 6.3°	39.2 ± 4.8°	1	0.090	0.3922 Small
HER (internal rotators)	64.8 ± 5.3°	62.2 ± 6.9°	4	0.057	0.3621 Small
HAB-HF9 (monoarticular adductors)	73.4 ± 5.8°	72.6 ± 4.8°	1	0.555	0.2209 Small
HF-KE (hamstrings)	74.0 ± 6.7°	74.4 ± 6.9°	0	0.716	0 No effect
KF (quadriceps)	115.4 ± 9.4°	113.8 ± 10.7°	2	0.428	0.2102 Small
HF-KF (gluteus maximus)	141.8 ± 6.2°	140.2 ± 4.9°	1	0.366	0.1961 No effect

Hip extension test [HE]; hip adduction with hip flexed 90° test [HAD-HF]; ankle dorsiflexion with knee flexed test [ADF-KF]; ankle dorsiflexion with knee extended test [ADF-KE]; hip abduction with hip neutral test [HAB]; hip internal rotation test [HIR]; hip external rotation test [HER]; hip abduction with hip flexed 90° test [HAB-HF]; hip flexion with knee extended test [HF-KE]; knee flexion test [KF]; hip flexion with knee flexed test [HF-KF]; The magnitude of the effect size of the pooled Standardised mean differences (SMD) was interpreted as trivial or no effect if SMD <0.2; small if SMD 0.2 to 0.59; moderate if SMD 0.6 to 1.19; large if SMD 1.20 to 2.00; very large if SMD 2.00 to 3.99 and extremely large if SMD greater than 4.00.

**Table 5:** Classification of a range of motion values (normal versus tightness) of the dominant limb in the 20 inline hockey players of the Spanish elite male and female national teams.

ROM		Tightness		Normal	R	*p*-Value
	n	Values	n	Values		
HE (iliopsoas)	18	6.2 ± 4.1°	2	20.0 ± 0.0°	−0.737	0.000
HAD-HF (piriformis)	11	24.7 ± 2.7°	9	32.2 ± 5.2°	−0.697	0.001
ADF-KE (gastronemius)	5	27.6 ± 3.8°	15	35.6 ± 5.2°	−0.595	0.006
ADF-KF (soleus)	16	37.2 ± 3.6°	4	47.0 ± 1.15°	−0.781	0.000
HAB (adductors)	0		20	40.2 ± 3.1°		
HIR (external rotators)	19	37.5 ± 6.0°	1	56.0°	−0.577	0.008
HER (internal rotators)	2	44.0 ± 5.7°	18	63.1 ± 7.3°	−0.642	0.002
HAB-HF (monoarticular adductors)	19	68.1 ± 7.8°	1	80.0°	−0.332	0.152
HF-KE (hamstrings)	20	72.4 ± 5.9°	0			
KF (quadriceps)	20	108.7 ± 11.5°	0			
HF-KF (gluteus maximus)	7	129.4 ± 4.4°	13	141.8 ± 4.6°	−0.809	0.000

Hip extension test [HE]; hip adduction with hip flexed 90° test [HAD-HF]; ankle dorsiflexion with knee flexed test [ADF-KF]; ankle dorsiflexion with knee extended test [ADF-KE]; hip abduction with hip neutral test [HAB]; hip internal rotation test [HIR]; hip external rotation test [HER]; hip abduction with hip flexed 90° test [HAB-HF]; hip flexion with knee extended test [HF-KE]; knee flexion test [KF]; hip flexion with knee flexed test [HF-KF]; r: Pearson’s r Correlation.

**Table 6:** Classification of range of motion values (normal versus tightness) of the non-dominant limb in the 20 inline hockey players of the Spanish elite male and female national teams.

ROM	Tightness		Normal		R	*p*-Value
	n	Value	n	Value		
HE (iliopsoas)	18	8.5 ± 3.9°	2	24.0 ± 5.7°	−0.770	0.000
HAD-HF (piriformis)	11	26.5 ± 4.9°	9	34.22 ± 3.2°	−0.688	0.001
ADF-KE (gastronemius)	5	25.2 ± 2.3°	15	36.0 ± 5.2°	−0.721	0.000
ADF-KF (soleus)	16	38.5 ± 3.5°	4	49.0 ± 2.0°	−0.805	0.000
HAB (adductors)	0		20	38.7 ± 3.3°		
HIR (internal rotators)	19	37.7 ± 4.2°	1	48.0°	−0.489	0.029
HER (external rotators)	2	41.0 ± 4.2°	18	59.6 ± 6.8°	−0.659	0.002
HAB-HF (monoarticular adductors)	19	68.5 ± 6.1°	1	80.0°	−0.398	0.082
HF-KE (hamstrings)	20	72.5 ± 5.5°	0			
KF (quadriceps)	20	108.9 ± 13.3°	0			
HF-KF (gluteus maximus)	7	128.0 ± 5.2°	13	138.8 ± 5.4°	−0.714	0.000

Hip extension test [HE]; hip adduction with hip flexed 90° test [HAD-HF]; ankle dorsiflexion with knee flexed test [ADF-KF]; ankle dorsiflexion with knee extended test [ADF-KE]; hip abduction with hip neutral test [HAB]; hip internal rotation test [HIR]; hip external rotation test [HER]; hip abduction with hip flexed 90° test [HAB-HF]; hip flexion with knee extended test [HF-KE]; knee flexion test [KF]; hip flexion with knee flexed test [HF-KF]; r: Pearson’s r Correlation.
